# Remembering the earthquake: intrusive memories of disaster in a rural Italian community

**DOI:** 10.1080/20008198.2022.2068909

**Published:** 2022-05-11

**Authors:** Alessandro Massazza, Helene Joffe, Elinor Parrott, Chris R. Brewin

**Affiliations:** a Department of Health Services Research and Policy, London School of Hygiene and Tropical Medicine, London, UK; b Department of Clinical, Educational and Health Psychology, University College London, London, UK

**Keywords:** Intrusive memories, PTSD, disaster, diary, 2016–2017 Central Italy earthquakes, Recuerdos intrusivos, TEPT, desastre, diario, Terremotos de 2016–2017 en el centro de Italia, 闯入性记忆, PTSD, 灾难, 日记, 2016–2017 年意大利中部地震

## Abstract

**Background:**

Disasters can have long-lasting impacts on mental health. Intrusive memories have been found to be common and persistent in the aftermath of earthquakes.

**Objective:**

To explore, using diaries, intrusive memories’ presence, content, characteristics, and relationship with probable post-traumatic stress disorder (PTSD) in a small rural community exposed to mass destruction and loss of life.

**Methods:**

Survivors of the 2016–2017 Central Italy earthquakes (*N* = 104) were first interviewed to investigate the presence of intrusive memories of the disaster. Those that reported intrusive memories were subsequently asked to complete a 7-day paper-and-pen diary tracking their spontaneous memories of the earthquake events.

**Results:**

Twenty months after the earthquakes, 49% (*n* = 51) of the sample reported having experienced intrusive memories post-earthquake and 38% (*n* = 39) reported at least one intrusive memory in their diaries. Memories were rated as being distressing, vivid, and experienced as a mixture of images and thoughts. The content of intrusive memories generally focused on sensations and experiences during the earthquake. Other common categories of content were the material environment and physical objects as well as human loss & death. Several memories had a social focus. A minority of memories contained more positive content as well as content from before and after the earthquake. Some participants (28%) experienced repeated intrusive memories of the same content. Memories of participants with and without probable PTSD did not significantly differ on characteristics or content.

**Conclusions:**

Intrusive memories can be common, distressing, and persistent occurrences following disasters, even in survivors not suffering from probable PTSD.

**Highlights:**

- Intrusive memories were common, distressing, and vivid more than 1-year post-disaster.
- They captured peri-earthquake sensations, material destruction, death, and social interactions.
- No difference in content or characteristics was found between participants with and without probable PTSD.

## Abstract (es)

**Antecedentes:** Los desastres pueden tener impactos duraderos en la salud mental. Se ha descubierto que los recuerdos intrusivos son comunes y persistentes después de los terremotos.

**Objetivo:** Explorar, a través de diarios, la presencia, el contenido, las características de memorias intrusivas y la relación con la probabilidad de trastorno de estrés postraumático (TEPT) en una pequeña comunidad rural expuesta a destrucción masiva y pérdida de vidas.

**Método:** Primero se entrevistó a los sobrevivientes de los terremotos de Italia central del 2016–2017 (*N* = 104) para investigar la presencia de recuerdos intrusivos del desastre. A aquellos que reportaron recuerdos intrusivos se les pidió posteriormente que completaran un diario con papel y lápiz por 7 días registrando sus recuerdos espontáneos de los eventos del terremoto.

**Resultados:** Veinte meses después de los terremotos, el 49% (*n* = 51) de la muestra informó haber experimentado recuerdos intrusivos posteriores al terremoto y el 38% (*n* = 39) informó al menos un recuerdo intrusivo en sus diarios. Los recuerdos se calificaron como angustiosos, vívidos y experimentados como una mezcla de imágenes y pensamientos. El contenido de los recuerdos intrusivos generalmente se centró en sensaciones y experiencias durante el terremoto. Otras categorías comunes de contenido fueron el entorno material y los objetos físicos, así como la pérdida y muerte humana. Varios recuerdos tenían un enfoque social. Una minoría de recuerdos contenía más contenido positivo, así como contenido de antes y después del terremoto. Algunos participantes (28%) experimentaron recuerdos intrusivos repetidos del mismo contenido. Los recuerdos de los participantes con y sin TEPT probable, no diferían significativamente en características o contenido.

**Conclusiones:** Los recuerdos intrusivos pueden ser experiencias comunes, angustiosas y persistentes después de los desastres, incluso en sobrevivientes que no sufren de TEPT probable.

## Abstract (zh)

**背景：**灾害会对心理健康产生长期影响。已发现闯入性记忆在地震后常见且持续存在。

**目的：**在一个遭受大规模破坏和生命损失的小型农村社区中，使用日记探索闯入性记忆的存在、内容、特征以及与可能的创伤后应激障碍 (PTSD) 的关系。

**方法：**首先访谈了 2016–2017 年意大利中部地震的幸存者（*N* = 104），以考查是否存在对灾难的闯入性记忆。那些报告闯入性记忆的人随后被要求完成一份为期 7 天追踪他们对地震事件自发记忆的纸笔日记。

**结果：**地震发生 20 个月后，49% (*n* = 51) 的样本报告在地震后经历过闯入性记忆，38% (*n* = 39) 在其日记中报告至少有一次闯入性记忆。记忆被认为是痛苦的、生动的，并且被体验为是图像和思想的混合体。闯入性记忆的内容通常集中在地震期间的感觉和经历。其他常见的内容类别是物质环境和物理对象以及人员丧失和死亡。一些记忆具有社会焦点。少数记忆中包含更多积极内容，以及地震前后的内容。一些参与者 (28%) 经历了对相同内容的重复闯入性记忆。有、无可能 PTSD 的参与者的记忆在特征或内容上没有显著差异。

**结论：**闯入性记忆在灾难后可能是常见的、痛苦的和持续发生的事，即使在不患有可能PTSD 的幸存者中也是如此。

Although disasters are known to be associated with increased rates of mental health problems in the exposed population (Goldmann & Galea, 2014), few studies have specifically examined how survivors experience intrusive memories in the long-term aftermath of such events. After earthquakes, for example, inhabitants may remain in situ after living through devastating levels of material damage and loss of life. These circumstances would be expected to influence the prevalence and nature of symptoms such as re-experiencing the traumatic event. Repeated re-experiencing of a small number of traumatic scenes is regarded as a hallmark of posttraumatic stress disorder (PTSD) (Bar-Haim et al., 2021). However, memory research would suggest that re-experiencing the traumatic event would be common in the presence of daily reminders of an emotionally intense experience (Brewin, 2014). Thus, in specific contexts re-experiencing of the traumatic event might no longer differentiate individuals with PTSD from those without. This would have important implications for the diagnosis and management of survivors.

The 2016–2017 Central Italy earthquake sequence involved extensive destruction wrought on a very small community. The earthquake sequence consisted of four major earthquake shocks (24 August 2016: 6.0 M_w_; 26 October 2016: 5.9 M_w_; 30 October 2016: 6.5 M_w_; 18 January 2017: 5.5 M_w_). The Italian regions most affected by the earthquake sequence were Latium, Umbria, Abruzzo, and Marche. The earthquake sequence led to the displacement of approximately 50,000 people and to widespread economic damage. The deathliest earthquake shock (299 causalities) took place at 3.36am on the night of the 24 August 2016 with epicenter at Accumoli, Latium. Most deaths, 239 out of 299, were recorded in the small village of Amatrice, an isolated rural community in the Apennines. As the total population of Amatrice pre-earthquake amounted to 2,646 individuals (Istituto Nazionale di Statistica, 2011), the mortality rate of almost 10% left everyone with a major loss. Additionally, the material destruction was extensive, with the village destroyed and its surrounding hamlets heavily damaged. Most of the population were homeless and living in campers or containers for months, until they were relocated in temporary housing shelters built by the State in the same area. To date, most inhabitants still reside in these temporary shelters as the reconstruction efforts and debris removal proceed at a slow pace.

Studies using retrospective questionnaires have suggested that intrusive symptoms are common and distressing occurrences following disasters, often persisting for months or years following the original event. A study conducted six months following the L’Aquila earthquake reported that 58.2% of the sample experienced intrusive symptoms (Roncone et al., 2013). Substantial intrusive symptomatology was still present 15–18 months following this disaster (Cofini, Carbonelli, Cecilia, Binkin, & di Orio, 2015). Similar findings have been reported following earthquakes in China (Tian, Wong, Li, & Jiang, 2014) and Turkey (Eksi & Braun, 2009).

Most studies to date have relied on questionnaires that required participants to report on how often they experienced the symptom or how distressing the experience of the symptom was over time (e.g. weeks or months). Among the limitations noted with this approach are the reliance on memory recall, poor understanding of the description of the symptom leading to imprecise answers, possible ceiling effects, and issues concerning over – and under-estimation of symptoms (Schuler et al., 2021). Questionnaires also reveal little about the content and characteristics of respondents’ intrusive memories, for example the variety in the themes and whether they are repeated. One way to assess this symptom more precisely is by using diaries in which participants are asked to report regularly on their symptoms over short time spans (Chun, 2016).

Diaries have been used to track intrusive memories naturalistically in samples of individuals exposed to trauma (Kleim, Graham, Bryant, & Ehlers, 2013; Kleindienst et al., 2017; Priebe et al., 2013; Schönfeld & Ehlers, 2017). However, aggregating data from participants exposed to different individual events is problematic as different types of event and degrees of exposure are likely to result in intrusive memories with different characteristics (Müller et al., 2016). Additionally, while some studies assessing intrusive memories from real-life traumatic events asked participants to report on the content of their intrusive memories (Kleim et al., 2013; Schönfeld & Ehlers, 2017), this qualitative information was generally not analyzed in depth. This content may nevertheless be important in advancing our understanding of re-experiencing (Hackmann, Ehlers, Speckens, & Clark, 2004). This study therefore represents the first to assess with diaries the presence, phenomenology, and content of intrusive memories experienced more than one year following trauma in a small community exposed to the same disaster.

The aims of the current study were to: (i) provide a description of prevalence, characteristics, and phenomenology of intrusive memories in a sample of disaster survivors still living in a post-disaster context more than one year following the event; (ii) explore the relationship between characteristics of intrusive memories and PTSD symptoms; and (iii) test the use of the diary methodology for intrusive memories in a post-disaster context.

## Methods

### Participants and recruitment

All participants were survivors of the 2016–2017 Central Italy earthquakes and most (88%) lived in Amatrice, with a minority (12%) residing in adjoining villages. Participants were identified based on a previous study conducted in the region by the authors (Massazza, Joffe, & Brewin, 2019). The local health services and municipality supported the authors in identifying participants for the research study. Participants were contacted either by phone or face to face by the first author who informed them of the study and invited them to participate. Participants were recruited from the general population that resided in the area when the earthquakes took place. A purposive sampling strategy was used to achieve a sample that reproduced the approximate demographic distribution of the population of Amatrice in terms of age and gender as per 2011 census (Istituto Nazionale di Statistica, 2011). Participants in the current study are a subset of a larger sample size that participated in another study by the authors (more information on recruitment and response rate for this sample reported in Massazza, Joffe, Hyland, & Brewin, 2021).

### Measures

Participants who reported having experience of post-earthquake intrusive memories during the initial interview were asked to complete a pen-and-paper daily diary for 7 days. The diary structure was adapted from Hørlyck, Bisby, King, and Burgess (2019). When experiencing an intrusive memory related to the earthquake events, participants were asked to enter the intrusion in the diary by providing a brief description of its contents. Additionally, participants were asked to report whether they experienced the memory as an image, a thought, or as mixture of both. They rated on a scale from 0 (*not at all*) to 5 (*extremely*) how distressing and vivid each memory was.

Participants also completed the PTSD Checklist for DSM-5 (PCL-5; Weathers et al., 2015). The PCL-5 is a 20-item self-report questionnaire assessing PTSD symptoms. The scale ranges from 0 (*not at all*) to 4 (*extremely*), focusing on how distressed the individual was by the symptoms over the last month. The PCL-5 has been shown to have high total internal reliability (*α* = .90) (Sveen, Bondjers, & Willebrand, 2016). The total internal reliability in the current sample was excellent (*α* = .91). The PCL-5 went through a thorough back-translation procedure to produce an Italian version.

Finally, demographic information was collected from the participants together with information on trauma exposure and material loss.

### Procedure

An interview was first conducted with participants to assess whether they had persistently experienced intrusive memories of the earthquake events in the months following the disaster. The first author read out a description of what an intrusive memory is (standard script adapted from Hackmann et al. (2004) and Evans, Ehlers, Mezey, and Clark (2007), reported in full in Supplementary Materials). If the participant reported having experienced intrusive memories, they were asked to focus on the most distressing one and provide a detailed description of its contents (Massazza, Joffe, & Brewin, 2021). The PCL-5 was also administered during this interview session. Participants reporting intrusive memories were asked to track these for 7 days using a paper-and-pen diary, starting from the day following the interview. The diary included a set of instructions which provided a reminder of the definition of intrusive memories, an explanation of what was meant by ‘thought’ and ‘image’, and an example detailing the differences between intrusive thoughts, intrusive images, and voluntary memories (adapted from Hørlyck et al., 2019). The first page of the diary was pre-filled as an example.

Participants were sent a daily reminder in the evening on their phones to complete the diary. If participants experienced no intrusive memories during a specific day, they were still instructed to make an entry in the diary specifying they had experienced zero memories. At the end of the 7 days, the diary was collected, and participants rated on a scale from 0 (*not at all*) to 10 (*extremely*) how compliant they had been in filling in the diary. The first author stressed the importance of answering this question honestly and that their answer would have no impact on their participation in the study or on the receipt of compensation (participants received 40€ as a reimbursement for their time). The mean compliance rate across participants was 7.46 (*SD* = 1.92).

Data collection took place for 3 months during May, June, and July 2018 (i.e. 20 months following the August 2016 earthquake). The UCL Research Ethics Committee approved the research. The project was also approved by the national health service centre of Rieti and by the local municipality, Comune di Amatrice. Prior to taking part, participants read an information sheet and provided written informed consent.

### Data analysis

We first investigated whether all entries in the diary would be eligible as intrusive memories of the earthquake events. Entries that did not include any content (e.g. entries describing the phenomenology of the memory such as ‘I remember as if zero time has passed’) or that were clearly not memories (e.g. anxious thoughts about possible future shocks such as ‘The fear that another earthquake could happen again’) were excluded. Two authors (AM & CB) agreed on the exclusion of 6 entries (reported in Supplementary Materials).

We then calculated the frequency of the memories across the seven days and their characteristics using descriptive statistics. We used a Pearson correlation to test the relationship between total number of intrusive memories reported and PCL-5 score. Due to violations of normality, Spearman correlation was used to assess the relationship between vividness and distress (distress and vividness for different memories were averaged across each participant). We then tested using Mann–Whitney U tests whether the memories of participants with probable PTSD (a cut-off point of >31 on the PCL-5 was used to determine positive PTSD status [Bovin et al., 2016]) scored differently on distress or vividness (similarly averaged across different memories for each participant).

We next assessed the proportion of participants that reported experiencing repeated intrusive memories, i.e. intrusive memories of the same content. Two authors (AM & CB) agreed on which memories could be labelled as repeated. We explored using independent-sample *t*-tests whether participants with repeated intrusive memories scored differently on the PCL-5.

Content analysis was used to record recurring categories of content in the memories. Content analysis has been defined as ‘establishing categories and then counting the number of instances in which they are used in a text or image’ (Joffe & Yardley, 2003, p. 58). This systematic method was chosen given the nature of the qualitative data, i.e. short descriptive sentences, and to allow for the quantification of qualitative data in the statistical analysis. Standard guidance for the conduct of content analysis was followed (Joffe & Yardley, 2003; Neuendorf, 2017). In practice, following immersion in the data, the authors agreed on inductive coding categories. An inductive approach was followed to allow exploration of novel content in the data. Next, one author applied the coding frame to each diary entry and determined whether the entry covered (entry assigned a ‘1’) or did not cover (entry assigned a ‘0’) a specific category*.* All memories were also categorized (a) according to whether they contained sounds, smells, or the shaking movements from the earthquake, and (b) whether they described moments taking place pre-earthquake, peri-earthquake (i.e. during the earthquake and immediately after), or post-earthquake. To ensure the reliability of the coding, a second coder (EP) blindly coded all content independently. We achieved an average Cohen’s kappa of .89 (range = .72–1.00) across the different categories,^1^ indicating almost perfect reliability (Altman, 1999). The few discrepancies identified were resolved. Differences in PCL-5 symptoms according to different categorizations were assessed using independent-sample t-tests.

## Results

### Participant characteristics

Out of 104 participants that were interviewed, a total of 51 (49%) participants reported experiencing intrusive memories in the aftermath of the earthquake. All 51 participants were asked to complete the diary. Of these, 9 participants were not included in the analyses as they reported no intrusions across the 7 days, 2 participants were excluded as they did not return their diary, and 1 participant was excluded as they refused to complete the diary (total number of participants excluded = 12). A total of 39 participants made at least one entry in the diaries and were therefore included in the analyses. Female participants comprised 59% (*n* = 23) of the sample, and mean age was 38.10 years (*SD* = 15.74). The mean score on the PCL-5 was 29.18 (*SD* = 15.67), 49% (*n* = 19) of participants exceeded the clinical cutoff score of 31. Thirteen participants (33%) had lost a close family member (e.g. child, parent), and 21 participants (54%) had lost a close friend. Most participants’ homes (87%) had been made uninhabitable^2^ by the earthquake resulting in most participants living in temporary housing.

### Memory characteristics

**Figure 1.**
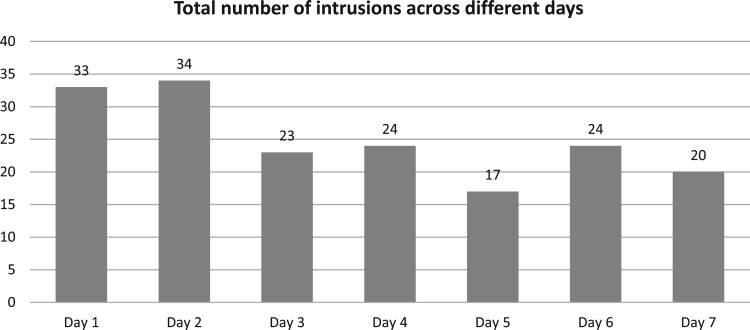
Total number of intrusive memories reported across the 7 days for all participants.

In total participants made 175 diary entries. The mean number of words in each diary entry was 7.17 (*SD* = 5.26, min = 1, maximum = 39). The number of intrusions experienced in each of the seven days is reported in Figure 1. The mean number of intrusive memories reported across the 7 days by each participant was 4.49 (*SD *= 2.49, min = 1, maximum = 10). No significant relationship was found between PCL-5 scores and total number of reported intrusive memories (*r* = .17, *p* = .304). Intrusive memories were more commonly experienced as a mixture of both image and thought (*n* = 73, 42%), with 53 being experienced only as images (30%), and 48 only as thoughts (28%). The mean level of distress associated with the intrusions was 3.59 (*SD* = 1.03) while the mean level of vividness was 3.84 (*SD* = 0.91). Distress and vividness, averaged within persons, were significantly correlated (*r*_s_ = .51, *p* = .001). No difference in mean distress (*z* = −.76, *p* = .448) or vividness (*z* = −.54, *p* = .593) was found between the memories of participants with and without probable PTSD.

### Memory categories

After reading through the whole set of memories, the authors agreed on establishing five categories of content: (1) Sensations & experiences during the earthquake; (2) Material environment and physical objects; (3) Human loss and death; (4) Social focus (i.e. involving the presence of other live people); and (5) Positive content. These five categories of content captured most memories (91%). An attempt was made to assign only one category to each memory, but a small minority of memories (*n *= 9, 5%) were assigned two categories when they clearly belonged to both (e.g. ‘The building in Amatrice that caused the most deaths’ [Male, 20] was assigned both the material environment and the human loss category). The frequency of each category is presented below in Table 1.

**Table 1.** Frequency of categories.

Category of content	Total number of memories	Frequency among participants
Sensations and experiences at the time of the earthquake	52	64% of participants
Material environment and physical objects	50	72% of participants
Human loss and death	32	59% of participants
Social memories	25	46% of participants
Positive content	10	21% of participants

The most common category of content was sensations and experiences at the time of the earthquake (*n* = 52, experienced by 64% of participants). These included descriptions of physical and emotional sensations experienced during the earthquake (e.g. ‘It’s dark and I am breathing dust’ [Female, 54], ‘*The earth shakes*’ [Female, 54], ‘Me being paralyzed and not understanding what was happening’ [Male, 61]) as well as descriptions of the shock (e.g. ‘Reliving the first moments of the shock’ [Female, 59]).

Fifty memories, experienced by 72% of participants, were concerned with the material environment and physical objects. Most of these memories (*n* = 32) focused on material destruction caused by the earthquake such as destroyed buildings or scenes of the destroyed town (e.g. ‘My collapsed shop’ [Female, 23], ‘My town destroyed’ [Male, 20], ‘The churches of Amatrice destroyed’ [Male, 20], ‘The objects that have remained in the debris of the demolished houses’ [Male, 20]). However, a number of these memories (*n* = 18) were not specifically concerned with material destruction but captured emotionally salient material environments (e.g. ‘My bedroom’ [Female, 31], ‘*My home*’ [Male, 25], ‘My elementary school’ [Male, 28], ‘The road to my home’ [Female, 33], ‘Memories of my town’ [Female, 26]) or objects connected to the disaster (e.g. ‘My torn pajamas’ [Female, 28], ‘A lot of balloons in the sky [during the State funerals]^3^’ [Female, 44]).

Thirty-two memories, experienced by 59% of participants, were concerned with human loss and death, such as seeing dead people or thinking about people that had died during the earthquake (e.g. ‘Extraction of one victim’ [Male, 70], ‘The faces of my deceased family members in front of the morgue camp’ [Male, 50], ‘People and friends that are not here anymore’ [Female, 50]). Additionally, a substantial number of memories (*n* = 25, experienced by 46% of participants) were social in nature (i.e. mentioned the presence of other live human beings) (e.g. ‘First person seen after the earthquake shock’ [Female, 33], ‘People asking for help’ [Male, 61]).

Finally, a minority of memories included content that was classified as being more positive in nature (*n* = 10, experienced by 21% of participants). Examples are ‘All the fantastic people that I got to know in this tragedy and that have remained close to me’ [Male, 51] and ‘Solidarity [of people after the earthquake]’ [Male, 49], and pleasant memories from the past of things that the earthquake had destroyed (e.g. a participant who had lost his children and his partner reported ‘Scenes from the daily life at home with my family’ [Male, 50], another participant reported how ‘Going through the Corso [the main road of the hamlet that was heavily damaged] brings to my memory moments of happiness and serenity walking my dog’ [Female, 33]). No significant differences in PCL-5 scores were found between participants who reported memories containing a specific category of content versus those who did not, largest *t*(37) = −1.26, *p* = .217.

Each participant reported an average of 3.97 different types of content across the 7 days (*SD* = 2.14, minimum = 1, maximum = 9). Some participants (*n* = 11, 28%) reported repeated content (i.e. the same memory intruding more than once). Most of these had a single repeated intrusion (*n* = 9), with two participants reporting two different repeated intrusions. Participants who reported experiencing repeated intrusions scored higher on the PCL-5 (*M* = 32.45, *SD* = 13.20) in comparison with participants without repeated intrusions (*M* = 27.89, *SD* = 16.58), but not significantly so (*t*(37) = −.81, *p* = .421).

### Sensory components

The most common sensory component of memories was visual, with 30% (*n* = 53) of all memories being classified as images (e.g. ‘Images of the people that died during the earthquake’ [Female, 33]). However, a smaller number of memories were also focused on sounds (*n* = 23, 13%), particularly the sound of the earthquake but also other sounds such as people screaming (e.g. ‘A deafening sound’ [Female, 54], ‘The screams of the people that were stuck under the debris’ [Male, 20]). Additionally, a few memories (*n* = 12, 7%) captured the physical feeling of shaking resulting from the earthquake shock (e.g. ‘Bed that shakes’ [Female, 23]). Only three memories focused on smells (2%) (e.g. ‘Smell of corpse’ [Female, 44]).

### Temporal dimension

Most memories captured content that had taken place during the peritraumatic phase, i.e. during the earthquake itself and in the immediate aftermath (*n* = 81, 46%). However, a minority of memories also contained content from the period after the earthquakes (*n* = 20, 11%) such as the funerals for the victims, life in temporary shelters, or the current state of destruction in the hamlets (e.g. ‘White coffins in a row the day of the State funerals’ [Female, 50], ‘The devastation in the hamlets that have been completely abandoned’ [Male, 49]). A minority of memories also captured content from periods prior to or immediately before the earthquake (*n* = 16, 9%) (e.g. a participant who had lost his child in the earthquake reported an intrusion of time spent together the day before his death, or ‘The last moments before the earthquake’ [Male, 28]). No significant differences on PCL-5 scores were found between participants who reported intrusions of any specific time period and participants who did not (largest *t*(37) = −2.01, *p* = .052, with participants who reported intrusive memories of peritraumatic moments reporting higher PCL-5 scores (*M *= 31.83, *SD* = 13.5) than those who did not (*M* = 20.33, *SD *= 19.5)).

## Discussion

This is the first study to provide a detailed assessment of intrusive memories in a sample of survivors who continue to inhabit the place where catastrophic levels of destruction and loss of life occurred. More than one year following the traumatic event, vivid and distressing intrusive memories were reported by more than a third of the sample. Although the presence (compared to the absence) of intrusive memories was associated with higher levels of PTSD (Massazza, Joffe, & Brewin, 2021), memories reported by those with and without probable PTSD were similar in their vividness and level of distress. This confirms findings on the prevalence and persistence of intrusive memories following disasters (Cofini et al., 2015; Roncone et al., 2013), and further suggests that in certain specific circumstances distressing intrusive memories are on their own not a reliable marker of PTSD.

A likely explanation for the high prevalence and long-term persistence of intrusive memories in this sample is that participants kept living in the same setting where the disaster took place and were confronted with everyday reminders of the event which could have functioned as triggers (Hackmann et al., 2004). The diary entry of one participant confirms this possible explanation by stating how ‘Passing through the Corso brings back to mind the chaotic moments of the morning of the earthquake’ [Male, 32]. Anecdotally, one participant wrote on the back of their diary how the only days when they did not experience intrusive memories were when they had left the town for work and were not confronted with daily material reminders. Future studies could compare the prevalence of intrusive memories in survivors who are relocated post-disaster versus those that remain in situ.

Among participants who completed the diaries, no significant associations between PTSD symptoms and the number or characteristics of intrusive memories were found. Although this may indicate that such memories are normative, it is also possible that we captured intrusive memories associated with other clinical conditions such as depression or prolonged grief disorder. For example, positive intrusive memories can occur as part of grief responses (Boelen & Huntjens, 2008). Intrusive memories have generally been shown to be transdiagnostic and to have differing phenomenological characteristics depending on the associated mental health condition (Brewin, Gregory, Lipton, & Burgess, 2010). Future studies assessing intrusive memories naturalistically in community samples should include a broader assessment of mental health outcomes.

A unique characteristic of this study is that the entire sample had been exposed to the same traumatic event, meaning that we could explore the range of common content. The most frequent category of content concerned sensations and experiences during the earthquake. Memories were often dominated by descriptions of the physical sensations experienced during the earthquake such as dust, darkness, loud noises, screams, and shaking. This confirms the highly sensorial nature of intrusive memories of trauma (Brewin et al., 2010). In general, most intrusive memories covered content that had taken place in the peritraumatic phase and participants who experienced intrusive memories of peritraumatic moments had higher rates of PTSD symptoms than those who did not (although this relationship only approached significance with *p* = .052). This adds to previous observations that the moments characterized by higher levels of peritraumatic reactions (e.g. dissociation) are more likely to be experienced as intrusions (Massazza, Joffe, & Brewin, 2021), and to cognitive theories of PTSD highlighting the importance of memory encoding during the peritraumatic phase (Brewin et al., 2010). A possible complementary explanation is that the commonality of intrusive memories of the earthquake shock was due to the frequency of earthquake-related triggers such as aftershocks taking place in the region and participants spending time in temporary structures with plastic floors that would remind them of earthquake-related shaking.^4^

When investigating the timeframe of intrusive memories, a minority of diary entries (8%) described intrusions concerning events taking place before the earthquakes, sometimes immediately before. These memories are more in line with the warning signal hypothesis of intrusive memories proposed by Ehlers et al. (2002). The fact that the earthquake occurred without warning while most inhabitants were asleep is likely to account for the small number of these intrusions. Finally, a minority of memories (11%) from the post-earthquake phase also intruded. These included memories of the funerals, memories of life in the tents in the weeks after the earthquake, aspects of daily life in a disaster setting such as eating in a communal canteen, and memories of the current state of abandonment of the hamlets. This highlights the prolonged nature of disaster stressors and the fact that, beyond the traumatic event *per se*, daily stressors in the recovery phase can also feature as distressing and vivid intrusive memories impacting mental health (Miller, Fernando, & Berger, 2009).

The second most frequently reported category of content concerned the material environment and physical objects. The loss and destruction of familiar spaces was at least as present in participants’ minds as the loss and destruction of human lives. The built environment may have been particularly present in their memories due to most participants having lost their homes and being daily exposed to debris of their town. This finding adds to the growing literature on the relationship between sense of place and wellbeing in the aftermath of disasters (Knez et al., 2018) and highlights the importance of including considerations around shelter, place attachment, and place identity within disaster mental health responses.

These findings also provide a further argument for the need for efficient, respectful, and swift management of disaster waste (UN Environment Programme, 2013) and appropriate reconstruction efforts to diminish the potential triggers for survivors. However, the topic of debris removal was highly sensitive in the current context and characterized by heterogeneous emotional responses. Anecdotally, while widespread discontent was expressed around delays in removing debris and completing demolitions, debris was often imbued with contrasting emotions as the pain and sadness intertwined with the comfort and attachment to material reminders of a past life and deceased family members and friends.

A substantial number of memories were social and many captured interactions taking place during the peritraumatic phase. The other people included in these memories were usually members of the community in distress (e.g. ‘My neighbour that night was escaping with wet pajamas because he had peed on himself because of fear’ [Female, 59]) or close family members (e.g. ‘The screams of XXX [partner] on the phone’ [Female, 31]). These findings confirm that living through a disaster is frequently not just about individual survival but involves a socially shared experience (Drury, 2018; Massazza, Brewin, & Joffe, 2021). Additionally, a minority of social memories featured people that had died during the earthquake when they were still alive (e.g. ‘*XXX* [deceased son] while we were exiting the hospital [day before earthquake]’ [Male, 50]), a type of memory characteristic of a grief-based response (Boelen & Huntjens, 2008).

While the social nature of intrusive memories might have been heightened in the current sample due to the small community, the presence of other people is common across many types of traumas. However, although the social and interpersonal content of intrusive memories has received some attention in the context of disorders such as social anxiety (Ashbaugh, Fishman, & Houle-Johnson, 2019) and bipolar disorder (Gregory, Brewin, Mansell, & Donaldson, 2010), it has remained largely unexplored within psychotraumatology. As a growing number of models stressing the social nature of trauma emerge (Maercker & Hecker, 2016), future research should investigate more systematically the presence or absence of social content within intrusive memories of traumatic events and its relationship with post-trauma psychopathology.

The study has several limitations. Firstly, the small sample size may have impacted the power of our statistical calculations and our results should be replicated using a larger sample. However, comparisons of distress and vividness in those with and without probable PTSD were based on the average of multiple observations, improving their reliability. Furthermore, we did not formally validate the PCL-5 measure of PTSD for our context, although its internal reliability was high in the current study. The use of a self-report instrument to assess probable PTSD status represents a further limitation. Moreover, our study is also limited by the lack of data on covariates that may have confounded the relationship between intrusive memories’ characteristics and PTSD symptoms (e.g. previous psychiatric history and current mental health comorbidities).

Additionally, while the use of a diary substantially reduces the risk of bias in retrospective accounts of symptoms, it still does not provide real-time monitoring of intrusive memories. Ecological momentary assessment can address this limitation and has been used to assess intrusive memories (Chun, 2016; Kleim et al., 2013) as well as mental health among disaster survivors (Dornbach-Bender et al., 2020). One additional possible limitation is that the diary itself and the daily reminders might have worked as triggers for the intrusive memories, therefore artificially increasing the number of reported intrusive memories. However, there is little evidence to date of measurement reactivity in diary tasks in general (Barta, Tennen, & Litt, 2012), or specifically in the context of tracking PTSD symptoms (Naragon-Gainey, Simpson, Moore, Varra, & Kaysen, 2012). Future research on intrusive memories should assess this risk more systematically. Furthermore, the need to write down one’s responses in a diary may have contributed to short entries and future studies may want to explore less demanding methods (e.g. using voice recordings in electronic diaries) or ask participants to provide as much detail as possible.

A final limitation concerns the degree of compliance with the diary completion. Diaries are time-intensive for participants, and this might affect engagement with the task, especially in a disaster setting where participants might have various other priorities. Although the compliance rate was good, we did notice a slight downward trend in the reporting of intrusive memories with time (see Figure 1).^5^ This could indicate diminishing engagement with the task or fatigue, which have been identified as possible issues in diary studies (Reynolds, Robles, & Repetti, 2016).

The current study tracked spontaneous intrusive memories using a diary in a sample of survivors exposed to the same disaster. Intrusive memories were present in a substantial proportion of survivors more than one year following the event and were equally vivid and distressing in participants with and without probable PTSD. These observations throw new light on the reality of continuing to inhabit the scene of a major disaster and suggest the need for further research to evaluate how recovery, and the reconstruction process, continue to impact psychologically on survivors. The findings also raise important questions about the value of intrusions as a marker of PTSD under such circumstances.

## Supplementary Material

Supplemental Material

## Data Availability

The data will be not publicly available due to information that may compromise the privacy of research participants present in the qualitative description of their intrusive memories.

## Notes

1 Sensations and experiences during the earthquake (*κ *= .81, 95% CI: .71–.91, *p* < .001); material environment & objects (*κ *= .91, 95% CI: .86–.98, *p* < .001); human loss (*κ *= .96, 95% CI: .88–1.00, *p* < .001); social focus (*κ *= .98, 95% CI: .93–1.02, *p* < .001); positive content (*κ *= .88, 95% CI: .72–1.04, *p* < .001); sound-based memory (*κ *= .98, 95% CI: .92–1.02, *p* < .001); shaking (*κ *= .95, 95% CI: .86–1.04, *p* < .001); smell-based memories (*κ *= 1.00, *p* < .001); pre-earthquake content (*κ *= .75, 95% CI: .57–.93, *p* < .001); peri-earthquake content (*κ *= .80, 95% CI: .71–.89, *p* < .001); post-earthquake content (*κ *= .74, 95% CI: .58–.90, *p* < .001).

2 We used the outcome of the AeDeS (Agibilità e danno in emergenza sismica) form to determine the status of participants’ houses. AeDes forms represent an assessment conducted by a technical professional on the compliance with safety standards of a building. All outcomes below A indicate different degrees of damage that make the house uninhabitable.

3 When content is inserted in [] it indicates an addition from the author to clarify the entry.

4 This was consistently noticed as all interview assessments took place in a temporary health facility built out of containers with plastic floors that would vibrate when people walked in the structure and participants often commented on how this physical sensation was like that experienced during the earthquake.

5 The use of an electronic diary would have allowed for a more precise understanding of the level of engagement with the diary throughout the 7 days, but the authors opted for a paper-and-pen diary due to the proportion of elderly participants who might have been less familiar with the use of electronic diaries.
