# Supplementary material for: Remembering the earthquake: intrusive memories of disaster in a rural Italian community
Source: Eur J Psychotraumatol. 2022 May 11;13(1):2068909. doi: 10.1080/20008198.2022.2068909 (PMC9103703; doi:10.1080/20008198.2022.2068909)
Supplement: Supplemental Material [file ZEPT_A_2068909_SM8385.docx]

**Supplementary Materials**

Supplement 1: Script presented to participants for intrusive memory identification

The following standard introduction adapted from Evans, Mezey, Ehlers & Clark (2007) was presented to participants to identify the presence of intrusive memories:

“*People who have gone through a distressing event can remember the event in different ways. Some people have memories of part of the earthquake that pop into their mind when they do not want them to. These are usually from particular moments from before, during or after the event that somehow “got stuck” in memory and keep coming back. These memories consist of part of what actually happened at the time and we will call them intrusive memories. Do you sometimes get or have got in the past such unwanted recollections of the earthquake?”.*

Supplement 2: Excluded memories

1. The continuous thought of new shocks and the terror that walks next to me
2. The fear that another earthquake shock could happen again
3. I remember as if zero time had passed
4. The memory is always alive even when I tell it to people I know or colleagues
5. Also when I am watching the news the memory confronts itself with my memories
6. Only images, as if of a surreal movie, but at the same time they are true images
